# Ginsenoside Rg5 increases cardiomyocyte resistance to ischemic injury through regulation of mitochondrial hexokinase-II and dynamin-related protein 1

**DOI:** 10.1038/cddis.2017.43

**Published:** 2017-02-23

**Authors:** Yi-Lin Yang, Jia Li, Kang Liu, Lei Zhang, Qun Liu, Baolin Liu, Lian-Wen Qi

**Affiliations:** 1 State Key Laboratory of Natural Medicines, China Pharmaceutical University, Nanjing, China; 2 Jiangsu Key Laboratory of TCM Evaluation and Translational Research, Department of Pharmacology of Chinese Materia Medica, China Pharmaceutical University, Nanjing, China

## Abstract

Hexokinase-II (HK-II) and dynamin-related protein 1 (Drp1) regulate mitochondrial function differently. This study was designed to investigate the cardioprotective effect of ginsenoside Rg5 (Rg5) with emphasis on the regulation of mitochondrial HK-II and Drp1. Saturated acid palmitate (PA) stimulation increased lactate accumulation and induced cellular acidification by impairing the activity of pyruvate dehydrogenase (PDH) in cardiomyocytes, leading to HK-II dissociation from mitochondria. Rg5 improved PDH activity and prevented cellular acidification by combating fatty-acid oxidation, contributing to protecting mitochondrial HK-II. HK-II binding to mitochondria prevented mitochondrial Drp1 recruitment, whereas Drp1 activation decreased the content of mitochondrial HK-II, demonstrating the reciprocal control for binding to mitochondria. Rg5 promoted Akt translocation to mitochondria and increased HK-II binding to mitochondria while coordinately suppressing Drp1 recruitment and mitochondrial fission. Akt inhibitor triciribine or knockdown of Akt with small interfering RNA diminished the effects of Rg5, indicating that Rg5 inhibited Drp1 activation and promoted HK-II mitochondrial binding through Akt activation. Rg5 prevented the opening of mitochondrial permeability transition pore and increased ATP production, resultantly increasing cardiomyocyte resistance to hypoxia/reoxygenation injury. Meanwhile, Rg5 prevented cell apoptosis with increased HK-II binding and reduced Drp1 recruitment to mitochondria in isoproterenol-induced ischemic heart of mice. Taken together, these findings not only established a previously unrecognized role of ginsenosides in cardioprotection but also suggest that mitochondrial HK-II binding and Drp1 recruitment could be targeted therapeutically to prevent ischemic injury in the heart.

The heart has a very high energy demand and uses fatty acid and glucose as energy substrates. Although the heart prefers fatty-acid oxidation for energy production, glucose is an efficient energy supply because the amount of ATP produced per O_2_ consumed is greater in glucose oxidation compared with fatty-acid oxidation.^1^ Fatty-acid oxidation and glucose oxidation compete for substrates, and the reciprocal control between fatty-acid and glucose metabolism is described as the Randle Cycle.^2^ Increased myocardial fatty-acid oxidation is often observed in patients with diabetes mellitus and obesity owing to the elevated circulating fatty acids and insulin resistance.^3, 4, 5^ In mitochondria, enhanced fatty-acid oxidation decreases glucose oxidation by inhibiting the activity of pyruvate dehydrogenase (PDH), the rate-limiting enzyme linking glycolysis to glucose oxidation, and then impairs cardiac energy efficiency, especially during ischemia/reperfusion (I/R).^6, 7^ The alternation of mitochondrial oxidation in the heart is considered the main cause for the vulnerability to ischemic injury in metabolic disoreders.^7^

Hexokinase (HK) catalyzes the first step in glycolysis by converting glucose to glucose 6-phosphate, and HK-II is the predominant isoform in the heart. HK-II enhances aerobic glycolysis in tumor cells (the Warburg effect) and thereby provides tumor cells with resilience to cell death.^8^ In cardiomyocytes, HK-II dynamically shuttles between the mitochondria and cytoplasm in response to cellular stress. Hence dysregualtion of mitochondrial HK-II has a critical influence on the susceptibility of the heart to I/R injury.^9^ HK-II binds to outer mitochondrial membrane via connection with voltage-dependent anion channel 1 (VDAC), which interacts with the adenine nucleotide translocase (ANT), forming a contact site between the outer and inner membranes.^10^ This complex is essential for the permeability barrier of the inner mitochondrial membrane (IMM) and the mitochondrial permeability transition pore (mPTP) acts as a key nodal point in mediating mitochondrial function. Historically, it was suggested that the mPTP was comprised of VDAC in the outer mitochondrial membrane and ANT in the IMM. In the past decade, however, genetic studies demonstrate that ANT and F_1_F_0_ATP synthase are the core constituents of the complex, responsible for mitochondrial homeostasis and energy generation.^11^ Moreover, a recent study demonstrates the implication of cardiac cAMP signaling in mitochondrial permeability transition.^12^ Loss of the permeability barrier of IMM is considered the final event causing cell death during I/R injury.^13^ HK-II detachment from mitochondria induces a conformational change of the molecular complex, leading to mitochondrial depolarization and cell death.^9, 14^ Therefore, mitochondrial HK-II dissociation during ischemia is correlated with cytochrome C release, ROS production and infarct size.^14^

Mitochondrial HK-II orchestrates metabolic and anti-cell death effects, contributing to mitochondrial homeostasis. Maintenance of functional and structural integrity of mitochondria is crucial for HK-II binding. Mitochondria are dynamic organelles and their morphological integrity is maintained by a delicate balance between mitochondrial fusion and fission. In response to ischemic stress, mitochondria undergo fragmentation, a fission process that is dependent on the activation of mitochondrial fission protein dynamin-related protein 1 (Drp1), resulting in mitochondrial dysfunction and apoptosis.^15^ In contrast, inhibiting mitochondrial fission prevents mPTP opening and reduces cell death.^9^ In the brain, mitochondrial fission occurs in ischemic stroke and precedes neuronal cell death.^16^ In addition, Drp1 activation and mitochondrial fission are demonstrated to be associated with endoplasmic reticulum stress, oxidative stress and mitochondrial dysfunction, which are mediators of I/R injury.^17, 18, 19^ Though the relation between Drp1 activation and mitochondrial HK-II binding is not known, we hypothesized that maintenance of the structural integrity of mitochondria should protect cardiomyocytes against I/R injury by increasing mitochondrial HK-II binding. In tumor cells, HK-II induction is mediated through Akt/mTORC1 pathway.^20^ In cardiomyocytes, HK-II has an Akt consensus sequence at position Thr473, which can be directly phosphorylated by Akt activation.^21, 22^ Mitochondria fission is mediated through Drp1 activation and inactivation of Drp1 by phosphorylating at Ser 637 residue is shown to prevent mitochondrial fission with its resultant consequences.^18, 19^ Because both HK-II and Drp1 exert their actions in mitochondria with opposite effects on cardiomyocyte survival, it is tempting to know whether HK-II and Drp1 regulate each other and compete for binding to mitochondria, especially during I/R injury.

*Panax ginseng* is a traditional herbal medicine used for enhancing human health and ginsenosides are the major bioactive constituents in ginseng root. Ginsenosides have been reported to promote glucose uptake in insulin-sensitive tissues, prevent ectopic lipid deposition^23, 24, 25^ and demonstrated potentially positive effects in heart diseases.^26^ Ginsenoside Rg5 (Rg5) is the most abundant compound in steamed ginseng.^27^ This study was designed to investigate if Rg5 promotes resistance to I/R injury in cardiomyocytes via regulation of energy metabolism and mitochondrial function. In the present study, we found that Drp1 activation impaired HK-II binding to mitochondria, setting cardiomyocytes at a stage susceptible to I/R injury. Rg5 inhibited Drp1 activation and preserved mitochondrial HK-II via Akt activation, thereby protecting the cell against I/R insult. These findings established a previously unrecognized role of ginsenosides in the regulation of mitochondrial function and suggest that mitochondrial Drp1 recruitment and HK-II binding could be targeted therapeutically to prevent I/R injury in the heart.

## Results

### Rg5 prevented acidification in cardiomyocytes

**Figure 1:**
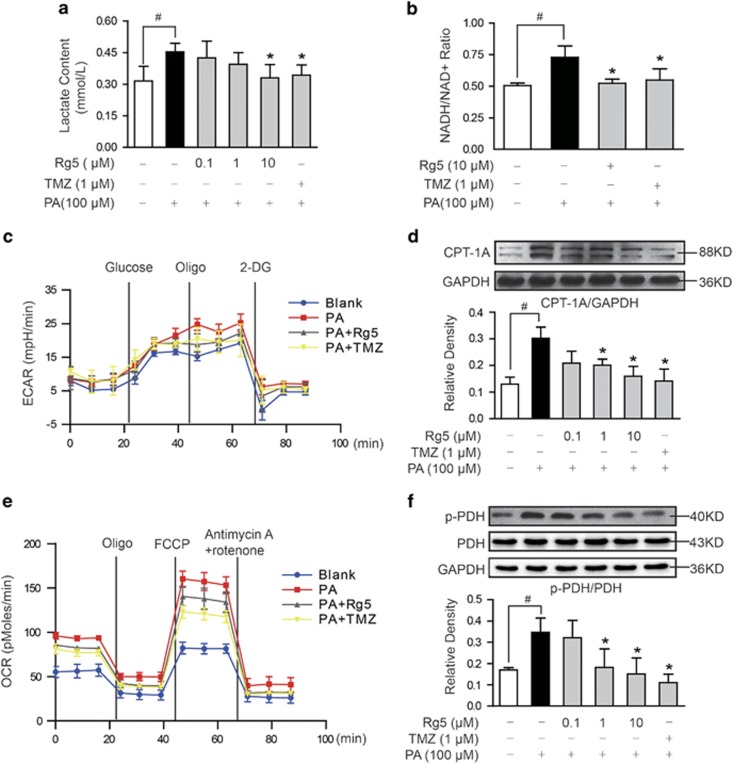
Rg5 prevented acidification in cardiomyocytes. Neonatal rat ventricular myocytes (NRVMs) were stimulated with palmitate (PA, 100 *μ*M) for 2 h. (**a** and **b**) Lactate contents (*n*=5) and NADH/NAD^+^ ratio (*n*=5). (**c**) Extracellular acidification ratio (ECAR) in NRVMs was measured using an XFe96 Extracellular Flux Analyzer (*n*=4). (**d**) CPT-1 protein expression in NRVMs was determined with western blot (*n*=3). (**e**) Oxygen consumption ratio (OCR) was measured using an XFe96 Extracellular Flux Analyzer (*n*=4). (**f**) Phosphorylation of PDH was detected with western blot (*n*=3). Data are expressed as mean±S.D. *P*<0.05 *versus* PA treatment; ^#^*P*<0.05 *versus* indicated treatment

Palmitate (PA) stimulation increased lactate accumulation and NADH/NAD^+^ ratio in neonatal rat ventricular myocytes (NRVMs), reflecting an increase in the cytosolic redox state (Figures 1a and b). Rg5 treatment limited lactate production, reduced NADH/NAD^+^ ratio (Figures 1a and b), and thereby prevented cellular acidification (Figure 1c). Carnitine palmitoyltransferase I (CPT-1) is a mitochondrial enzyme allowing fatty acids entry into mitochondria for oxidation. Rg5 prevented fatty-acid entry into mitochondria by downregulation of CPT-1 expression (Figure 1d) and reduced oxygen consumption ratio (OCR) (Figure 1e) in NRVMs, demonstrating its inhibitory effect on fatty-acid oxidation in mitochondria. PDH links glycolysis to glucose oxidation by converting pyruvate to acetyl-CoA for tricarboxylic acid cycle and thus shifts pyruvate from lactate to oxidation. Rg5 preserved PDH activity by dephosphorylation (Figure 1f), and this action should contribute to reducing lactate accumulation. Together, these results demonstrated that Rg5 reduced fatty-acid oxidation in mitochondria and prevented cellular acidification by reestablishing coupling of glycolysis to glucose oxidation. Trimetazidine, an anti-ischemic metabolic agent known as fatty-acid oxidation inhibitor, demonstrated a similar regulation as Rg5 (Figure 1).

### Rg5 prevented HK-II detachment from mitochondria

**Figure 2:**
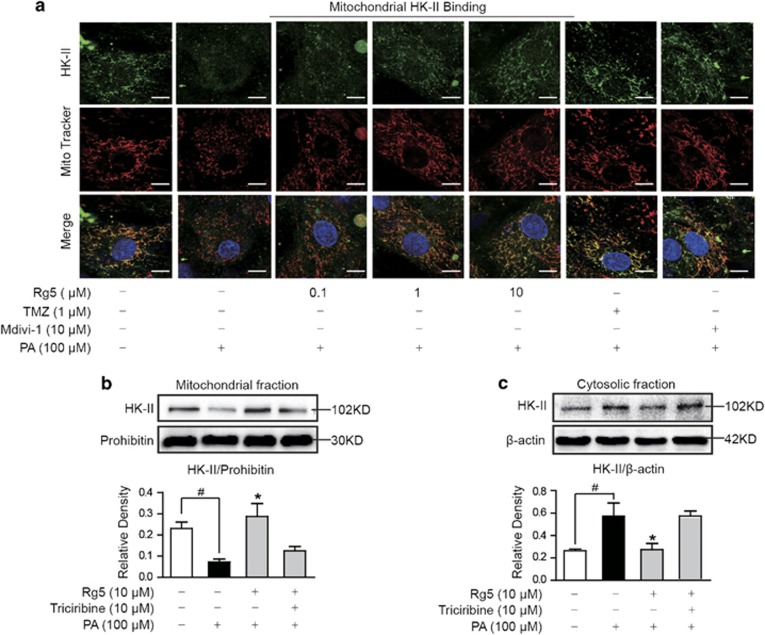
Rg5 prevented HK-II detachment from mitochondria. Neonatal rat ventricular myocytes (NRVMs) were stimulated with palmitate (PA, 100 *μ*M) for 2 h. (**a**) View of mitochondrial localization of HK-II with confocal scanning microscope (Green: HK-II; Red: MitoTracker Red cMXRos; Scale bars: 10 *μ*m); (**b** and **c**) Mitochondrial and cytosolic HK-II expressions were determined by western blot. Data are expressed as mean±S.D. from three or four independent experiments. *P<0.05 *versus* PA treatment; ^#^*P*<0.05 *versus* indicated treatment

Mitochondrial HK-II is susceptible to cellular acidification.^14^ Cardiac acidification induced HK-II to dissociate from mitochondria, but this alternation was prevented by Rg5 as well as trimetazidine treatment (Figure 2a). Mitochondrial fission inhibitor Midiv1 reduced HK-II detachment from mitochondria, suggesting the potential role of mitochondrial morphology in HK-II binding. Western blot showed that Rg5 restored HK-II expression in mitochondria and concomitantly reduced HK-II expression in the cytosol, further confirming that Rg5 prevented HK-II detachment from mitochondria in response to PA stimulation (Figures 2b and c). Akt inhibitor triciribine diminished the effect of Rg5 on the protection of mitochondrial HK-II, suggesting Rg5 increased HK-II association with mitochondria via regulation of Akt (Figures 2b and c).

### Rg5 promoted Akt association with HK-II in mitochondria

**Figure 3:**
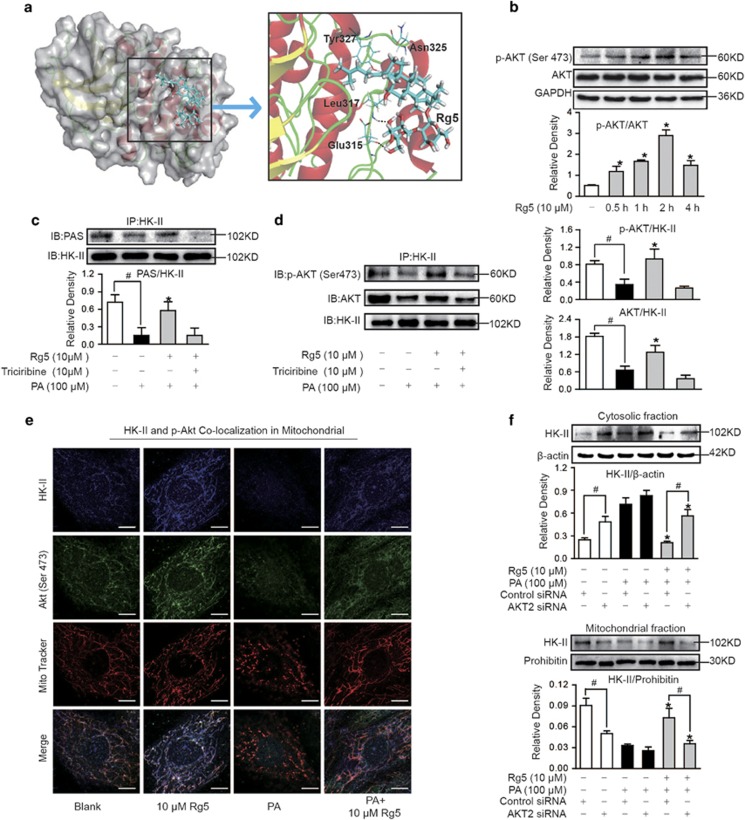
Rg5 promoted Akt association with HK-II in mitochondria. (**a**) Molecular docking analysis of the interaction between Rg5 and Akt. (**b**) Akt phosphorylation (S473) in neonatal rat ventricular myocytes (NRVMs) was determined by western blot. (**c** and **d**) NRVMs were stimulated with PA for 2 h, and then PAS, Akt or p-Akt (S473) in HK-II were determined with immunoprecipitation and western blot. (**e**) NRVMs were stimulated with PA for 2 h. Confocal image of HK-II and p-Akt (S473) colocalization in mitochondria were viewed with confocal scanning microscope (Blue: HK-II; Green: p-Akt; Red: MitoTracker Red cMXRos. Scale bars: 10 *μ*m); (**f**) H9c2 cells were transfected with Akt2 or control scrambled siRNA and then stimulated with palmitate (PA, 100 *μ*M) for 2 h. Mitochondrial and cytosolic HK-II expressions were determined using western blot. Data are expressed as mean±S.D. from three independent experiments. *P<0.05 *versus* control siRNA+PA treatment; ^#^*P*<0.05 *versus* the indicated treatment

To know if Rg5 regulated mitochondrial HK-II binding through Akt activation, we first performed molecular docking of Rg5 to Akt using the Autodock program. According to our docking result, Rg5 could bind to Akt by forming two stable hydrogen bonds with Glu 315 and Leu 317, as shown in Figure 3a. The binding of Rg5 probably induced the conformational changes of Akt, which would facilitate the phosphorylation at Ser 473. To validate the mimic analysis, we observed the effect of Rg5 in NRVMs, and found that Rg5 enhanced Akt phosphorylation (Ser 473), and significant effects were observed at 2 and 4 h (Figure 3b). Immunoprecipitation examination showed that phosphorylated Akt consensus sequence (PAS) presented in HK-II and Rg5 treatment increased PAS expression when cells were exposed to PA (Figure 3c). When HK-II was immunoprecipitated and then blotted using Akt or p-Akt (Ser 473) antibodies, Rg5 treatment increased total Akt and phosphorylated Akt expression in HK-II protein in cardiomyocyte (Figure 3d), indicating that Rg5 promoted Akt binding to HK-II. Consistently, confocal scanning further confirmed that Rg5 promoted Akt to translocate to mitochondria and join HK-II, indicated by the increased blue staining in the merged image (Figure 3e). When PA stimulation led to HK-II and Akt detachment from mitochondria, the alternation was prevented by Rg5 treatment (Figure 3e). We transfected H9c2 cells with small interfering RNA (siRNA) to silence Akt and found that the effect of Rg5 on the increased mitochondrial HK-II was diminished (Figure 3f), further confirming that Rg5 promoted HK-II binding to mitochondria though regulation of Akt activation.

### Rg5 inhibited Drp1 activation in cardiomyocytes

**Figure 4:**
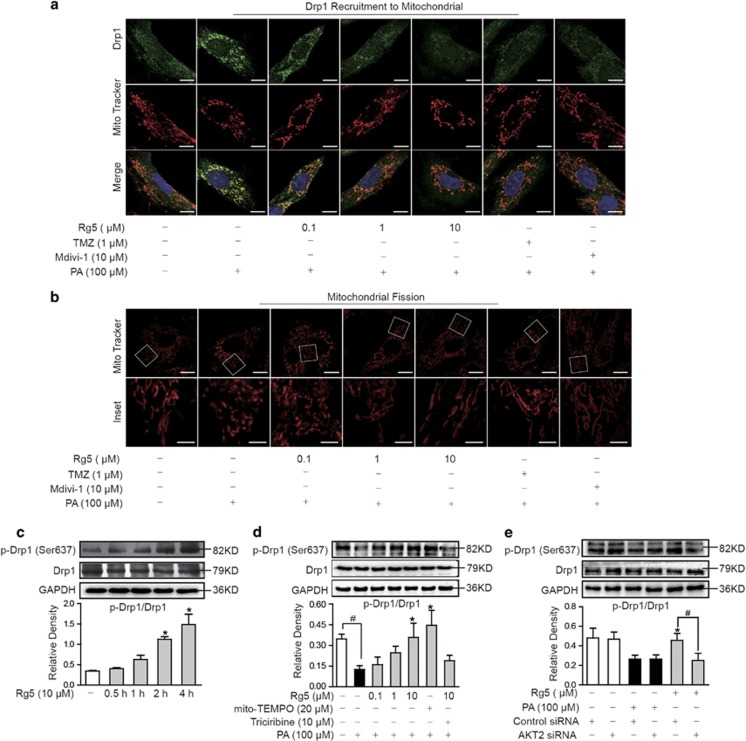
Rg5 inhibited Drp1 activation in cardiomyocytes. Neonatal rat ventricular myocytes (NRVMs) were stimulated with palmitate (PA, 100 *μ*M) for 2 h. (**a**) Confocal image of Drp1 in mitochondria (Green: Drp1; Red: MitoTracker Red cMXRos; Scale bars: 10 *μ*m). (**b**) Mitochondrial fission was viewed by Mito Tracker Red CMXRos with confocal microscopy (Upper, scale bar, 10 *μ*m; Lower, magnified images, bar, 5 *μ*m). (**c**) The phosphorylation of Drp1 (S637) in NRVMs was determined by western blot; *P<0.05 *versus* the control. (**d**) Drp1 phosphorylation (S637) in NRVMs treated with PA for 2 h. *P<0.05 *versus* PA treatment; ^#^*P*<0.05 *versus* indicated treatment. (**e**) Drp1 phosphorylation (S637) in H9c2 cells transfected with Akt2 or control scrambled siRNA was determined by western blot. Data are expressed as mean±S.D. from three independent experiments. *P<0.05 *versus* control siRNA+PA treatment; ^#^*P*<0.05 *versus* indicated treatment

We then investigated the effect of Rg5 on Drp1 activation in NRVMs. The view of confocal microscopy showed that upon PA stimulation, Drp1 recruited to mitochondria and caused mitochondrial fragmentation in NRVMs, whereas Rg5 treatment inhibited Drp1 recruitment and normalized mitochondrial structure (Figures 4a and b), indicating that Rg5 prevented mitochondrial fission by inhibition of Drp1 activation. Trimezivi-1 also inhibited Drp1 activation and prevent mitochondrial fission (Figures 4a and b). Phosphorylation of Drp1 at Ser 637 residue is proposed to inactivate Drp1.^16^ In NRVMs, Rg5 enhanced phosphorylation of Drp1 (Ser 637) and this regulation was preserved when cells were exposed to PA challenge, exerting the ability to suppress Drp1 activation (Figures 4c and d). Mito-TEMPO, a mitochondria-targeted antioxidant, also preserved Drp1 phosphorylation (Ser 637) in PA-stimulated NRVMs (Figure 4d). Akt inhibitor triciribine blocked Rg5 action in the regulation of Drp1 phosphorylation, indicative of the involvement of Akt activation in Rg5 action.

### Rg5 inhibited Drp1 activation via regulation of Akt

**Figure 5:**
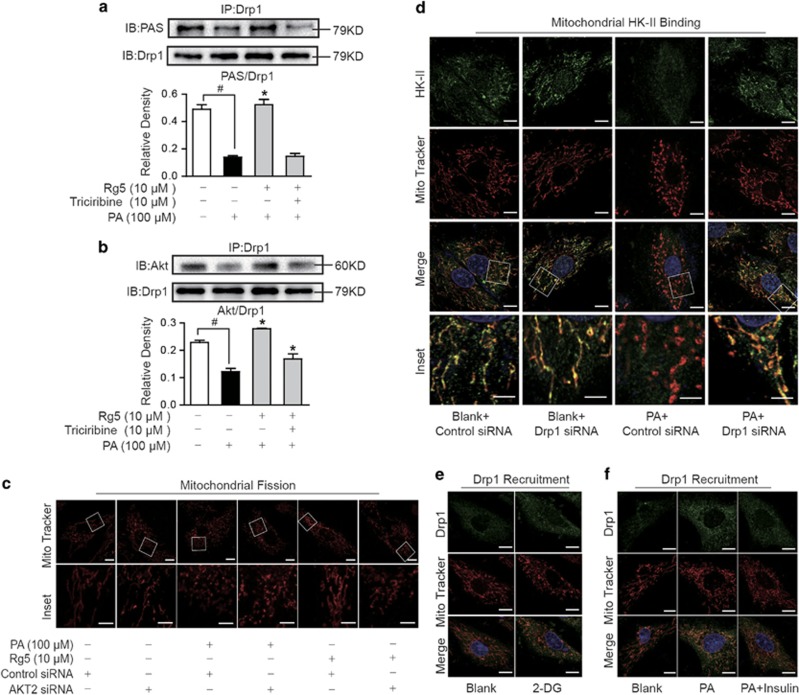
Regulation of Drp1 and HK-II in mitochondria. Neonatal rat ventricular myocytes (NRVMs) were stimulated with palmitate (PA,100 *μ*M) for 2 h. (**a** and **b**) Immunoprecipitation and western blot examination of PAS and Akt in precipitated Drp1 protein. *P<0.05 *versus* PA treatment; ^#^*P*<0.05 *versus* indicated treatment. (**c**) Confocal scanning image of mitochondrial fission (Mito Tracker Red CMXRos). Upper, scale bar, 10 *μ*m; Lower, magnified images, bar, 5 *μ*m. (**d**) Confocal scanning image of mitochondrial HK-II expression in H9c2 cells transfected with Drp1 or control scrambled siRNA (Green: HK-II; Red: MitoTracker Red CMXRos; Upper, scale bar, 10 *μ*m; Lower, magnified images, bar, 5 *μ*m) (**e** and **f**) Confocal scanning image of mitochondrial Drp1 expression in NRVMs pretreated with 2-deoxy-D-glucose (2-DG, 5 mM) for 24 h or insulin (0.1 *μ*M) 30 min (Green: Drp1; Red: MitoTracker Red cMXRos; Scale bars: 10 *μ*m). Data are expressed as mean±S.D. (*n*=3). The results are confirmed by three independent experiments

We prepared Drp1 protein using immunoprecipitation method from NRVMs and found that PAS presented in Drp1 protein (Figure 5a), suggesting the regulatory potential of Akt. Similar to the regulation of HK-II, Rg5 treatment increased PAS and Akt expression in Drp1 (Figures 5a and b), indicating that Rg5 promoted Akt location on Drp1. In H9c2 cells, siRNA knockdown of Akt did not influence mitochondrial structure, but diminished the inhibitory effect of Rg5 on mitochondrial fission in PA-stimulated cells, indicating that Rg5 protected mitochondrial morphological integrity dependent on Akt activation (Figure 5c).

To explore the potential interaction between Drp1 activation and mitochondrial HK-II, we observed mitochondrial HK-II in H9c2 cells when Drp1 was silenced by siRNA. PA stimulation reduced the content of HK-II in mitochondria and this change was blocked by knockdown of Drp1, indicating that inhibition of Drp1 activation could increase HK-II binding to mitochondria (Figure 5d). 2-deoxy-D-glucose (2-DG) and insulin are proposed to reduce or increase HK-II binding to mitochondria, respectively. To know if mitochondrial HK-II influenced Drp1 activation, we observed Drp1 recruitment in the presence of 2-DG or insulin, respectively. We found that treatment with 2-DG in NRVMs increased Drp1 recruitment to mitochondria, suggesting that the loss of mitochondrial HK-II promoted Drp1 recruitment to mitochondria (Figure 5e). In contrast, insulin prevented Drp1 recruitment to mitochondria in response to PA challenge (Figure 5f). Together, these results elucidated the reciprocal control between HK-II and Drp1 for the completion of mitochondrial binding.

### Rg5 ameliorated mitochondrial dysfunction in cardiomyocytes

**Figure 6:**
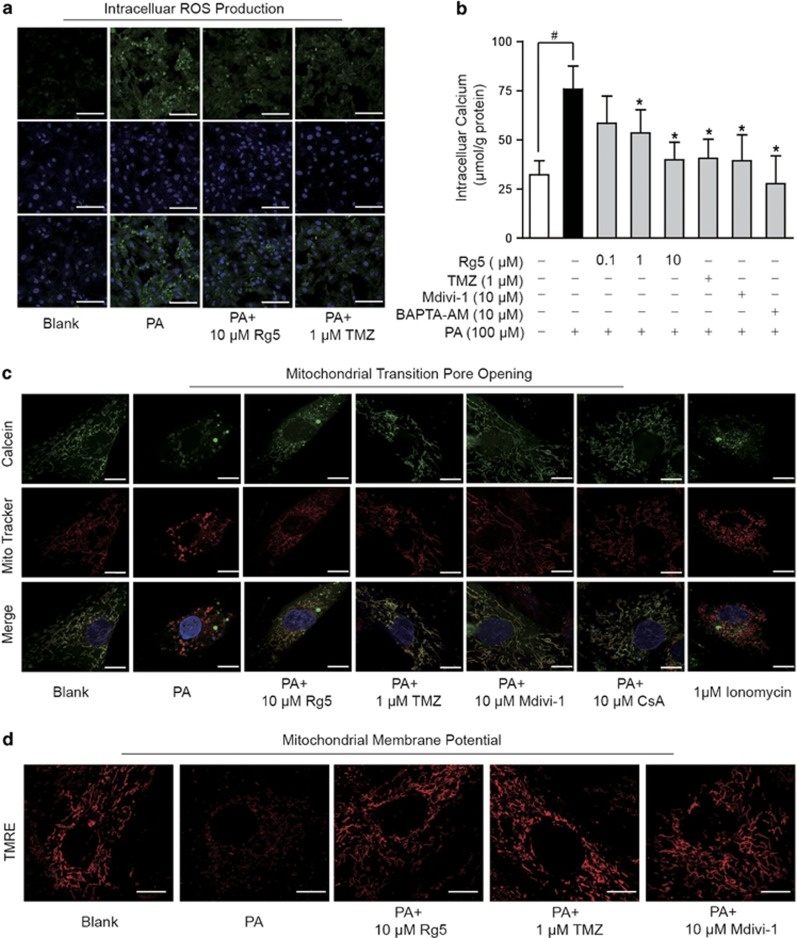
Rg5 ameliorated mitochondrial dysfunction in cardiomyocytes. Neonatal rat ventricular myocytes (NRVMs) were stimulated with palmitate (PA, 100 *μ*M) for 2 h. (**a**) Intracellular ROS production was viewed by dihydroethidium staining labeling with confocal microscopy. Scale bar, 100 *μ*m; (**b**) Intracellular calcium concentration. *P<0.05 *versus* PA treatment; ^#^*P*<0.05 *versus* indicated treatment; Data are expressed as mean±S.D. (*n*=4). (**c**) Calcein-AM combined with CoCl_2_ were used in viewing mitochondrial transition pore opening in cardiomyocytes. Scale bar, 10 *μ*m. (**d**) Mitochondrial potential (*Δψm*) was viewed by Tetramethylrhodamine ethyl ester perchlorate (TMRE) labeling with confocal microscopy. Scale bar, 10 *μ*m. The results are from three independent experiments

The direct consequence of mitochondrial HK-II disruption is mitochondrial dysfunction. In PA-stimulated NRVMs, mitochondrial ROS production (Figure 6a) and cellular calcium accumulation (Figure 6b) increased, indicative of oxidative stress and calcium overload (Figures 6a and b). These alternations were prevented by Rg5 treatment (Figures 6a and b). As a result of improved mitochondrial homeostasis, Rg5 prevented the opening of mPTP (Figure 6c) and restored the loss of the mitochondrial membrane potential (*Δψm*) (Figure 6d), demonstrating the protective effect on mitochondrial function. Trimetazidine and Mdivi-1 demonstrated a similar regulation as Rg5. As a calcineurin inhibitor, cyclosporine A prevented mPTP opening in NRVMs (Figure 6c). In contrast, calcium ionophore ionomycin induced mPTP opening (Figure 6c). The different regulations suggested the involvement of calcium overload in PA-induced mPTP opening.

### Rg5 increased cardiomyocyte resistance to I/R injury

**Figure 7:**
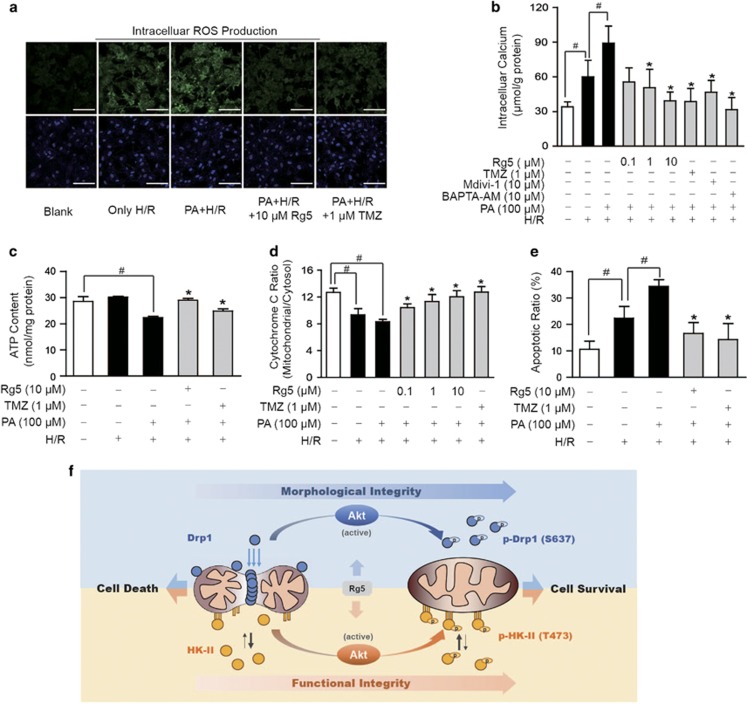
Rg5 increased cardiomyocyte resistance to I/R injury. Neonatal rat ventricular myocytes (NRVMs) were stimulated with palmitate (PA, 100 *μ*M) for 2 h before 4 h hypoxia (1% O_2_) and 1 h reoxygenation (H/R). (**a**) Intracellular ROS production in cardiomyocytes was viewed by dihydroethidium staining labeling with confocal microscopy. Scale bar, 100 *μ*m. The results are confirmed by three independent experiments. Intracellular calcium concentration (**b**) and ATP content (**c**) were detected by commercial kits (*n*=4). (**d**) The level of cytochrome C in mitochondrial and cytosol fraction were measured by ELISA (*n*=5). (**e**) The apoptosis assay was performed with AnnexinV/PI staining by flow cytometry analysis (*n*=3). Data are expressed as mean±S.D. *P<0.05 *versus* PA with H/R treatment; ^#^*P*<0.05 *versus* indicated treatment. (**f**) The proposed mechanism by which Akt activation prevented Drp1 recruitment and mitochondrial HK-II dissociation in cardiomyocyte

Rg5 inhibited Drp1 activation and promoted HK-II binding to mitochondria. Therefore, it was tempting to know whether these actions contributed to protecting cardiomyocytes from I/R injury. We pretreated NRVMs with PA in the presence of Rg5 for 2 h. After washing, cells were re-incubated under 1% O_2_ without glucose in the medium, followed by reoxygenation with glucose supply to mimic I/R insult. Oxygen/glucose depletion and reperfusion increased ROS production and calcium accumulation, whereas PA treatment augmented these responses (Figures 7a and b). Rg5 treatment suppressed ROS generation and reduced calcium accumulation (Figures 7a and b). As expected, Rg5 increased ATP content, prevented cytochrome C release from mitochondria and resultantly protected cells from apoptosis using flow cytometry analysis (Figures 7c–e). As a result, Rg5 promoted cardiomyocyte resistance to I/R injury by protecting the structural and functional integrity of mitochondria. Together with above-mentioned results, our work demonstrated that Rg5 promoted HK-II binding to mitochondria and prevented Drp1 recruitment via regulation of Akt activation, thereby protecting cardiomyocytes against ischemic insult. The proposed pathway was shown in Figure 7f.

### Rg5 protected mitochondrial integrity in isoproterenol-induced cardiac ischemia

**Figure 8:**
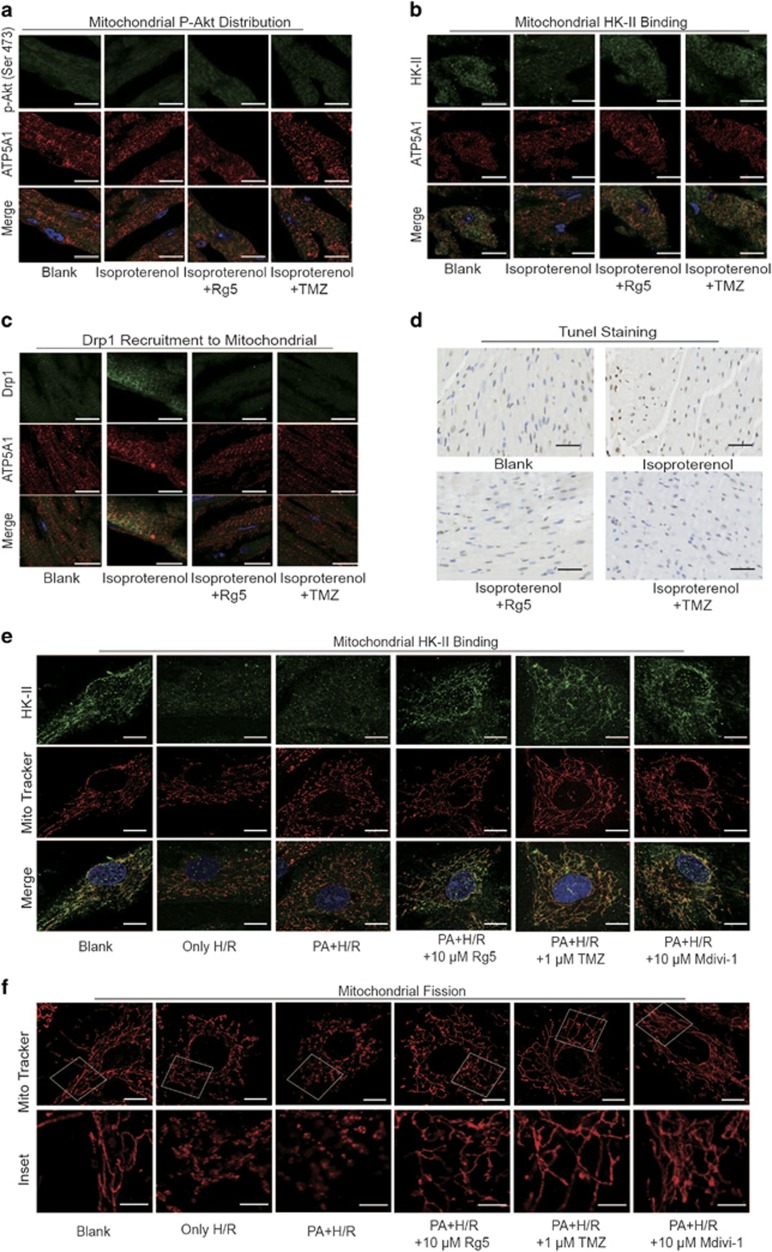
Rg5 protected mitochondrial integrity in isoproterenol-induced ischemic heart of mice. (**a**–**d**) Mitochondrial p-Akt distribution (**a**), HK-II binding (**b**) and Drp1 recruitment (**c**) in mitochondria were stained with immunofluorescence and viewed by confocal image (*n*=4). Scale bar, 10 *μ*m. (**d**) Heart paraffin section was stained with TUNEL (*n*=3–6). Scale bar, 50 *μ*m. Neonatal rat ventricular myocytes (NRVMs) were stimulated with palmitate (PA, 100 *μ*M) for 2 h before 4 h hypoxia (1% O_2_) and 1 h reoxygenation (H/R). Mitochondrial HK-II binding (**e**) and mitochondrial fission (**f**) were viewed by confocal image. Scale bar, 10 *μ*m. The results are confirmed by three independent experiments

To know the important role of mitochondrial integrity in the prevention of cardiac ischemic injury, we treated mice with isoproterenol to induce cardiac ischemia and observed the effect of Rg5 *in vivo*. Cardiac ischemia reduced Akt expression in cardiac mitochondria (Figure 8a), leading to the dissociation of HK-II from mitochondria (Figure 8b). Oral administration of Rg5 promoted Akt translocation to mitochondria and thus preserved mitochondrial HK-II (Figures 8a and b). Concordantly, Rg5 prevented Drp1 recruitment to mitochondria in the heart (Figure 8c). As a result of the protection of mitochondrial integrity, Tunnel staining showed that Rg5 reduced cell apoptosis in ischemic heart, demonstrating its protective effect on cardiomyocyte survival (Figure 8d). Meanwhile, we observed that Rg5 reduced the elevated levels of blood free fatty acids in isoproterenol-treated mice (Supplementary Figure), a regulation potentially contributing to attenuating the impact of fatty acid on cardiac metabolism.

In parallel, we observed the impact of saturated acid challenge on mitochondrial integrity during I/R insult in cardiomyocytes and found that pretreated PA reduced the content of mitochondrial HK-II and exaggerated mitochondrial fragments in response to hypoxia/reoxygenation insult (Figures 8e and f). Rg5 treatment during PA challenge increased HK-II binding to mitochondria and prevented mitochondrial fission when cells were exposed to 1% O_2_ and reoxygenation. These results further confirmed that maintenance of mitochondrial integrity was essential for resistance to ischemic injury.

## Discussion

HK-II binding to mitochondria protects cardiomyocyte survival by increasing energy efficiency. In contrast, Drp1 recruitment to mitochondria induces cell apoptosis through mitochondrial fission. In the current study, we found that HK-II and Drp1 competed for mitochondrial binding and their reciprocal control could be regulated by Akt. This finding suggests the potential role of Akt activation in cardioprotection.

Increased fatty-acid oxidation in mitochondria reduces carbohydrate oxidation by the uncoupling of glycolysis from pyruvate oxidation, and thus decreases cardiac efficiency in the use of energy.^7^ PDH converts pyruvate into acetyl-CoA, thereby being key in linking glycolysis to pyruvate oxidation. In the present study, saturated acid challenge increased fatty-acid oxidation in mitochondria accompanied by impaired PDH activity and lactate accumulation, indicating the inhibition of glucose oxidation by fatty-acid oxidation, a regulation also known as the Randle Cycle.^2^ As pyruvate oxidation is the major source for ATP production compared with glycolysis, the impaired glucose oxidation reduced heart energy efficiency. It has been well documented that PDH is inhibited by NADH and acetyl-CoA produced from fatty-acid oxidation.^28^ Rg5 reduced fatty-acid entry into mitochondria by downregulation of CPT-1 and then decreased oxygen consumption in mitochondria, demonstrating its inhibitory effects on increased fatty-acid oxidation in mitochondria. Moreover, we showed that combating fatty-acid oxidation improved PDH activity, and thereby re-established the coupling of glycolysis and glucose oxidation, indicating the important role of PDH activity in the control of intracellular redox homeostasis.

It is generally considered that HK-II detachment from mitochondria mainly occurred during cardiac ischemia.^29^ Herein we showed that increased fatty-acid oxidation weakened HK-II binding to mitochondria owing to increased redox state, causing the enzyme to translocate to the cytoplasm. Rg5 inhibited fatty-acid oxidation in mitochondria and reduced lactate accumulation by restoring PDH activity, and thereby prevented HK-II dissociation from mitochondria. These findings indicate that the alternation of mitochondrial oxidation is an important cause for HK-II detachment from mitochondria.

Tumor cells show increased Akt activity which facilitates tumor survival through upregulation of HK expression. In tumor cells, HK-II expression and binding to mitochondria are regulated by Akt/mTOR pathway.^10, 20^ Therefore, the specific property in tumor survival raises the possibility that pharmacological activation of Akt might protect cardiomyocyte survival by increasing mitochondrial HK-II binding. Consistent with this, it is documented that Akt activation prevents cardiomyocyte apoptosis induced by multiple pathological insults including ischemia reperfusion.^30, 31^ Rg5 activated Akt in cardiomyocytes and induced Akt consensus PAS induction in HK-II protein, suggesting the possibility that Rg5 regulates mitochondrial HK-II through Akt activation. Akt traverses the cell interior with regulated localization, and consistent with this, we found that Rg5 treatment promoted Akt translocation to mitochondria and together with HK-II increased mitochondrial binding (Figure 3e). Akt inhibitor triciribine and knockdown of Akt blocked Rg5 action in the protection of mitochondrial HK-II, further confirming that Rg5 increased HK-II binding to mitochondria via regulation of Akt activation. These results were consistent with a previous study showing that Akt increases mitochondrial HK-II association by phosphorylation of HK-II.^22^

The location of Akt in subcellular compartments is a key determinant of its biological effects. In addition to the regulation of HK-II, we found that Rg5 treatment induced PAS induction and increased activated Akt expression in Drp1 protein, indicating another molecular target regulated by Akt in mitochondria (Figures 5a and b). In response to activation, the cytoplasmic Drp1 translocates to the mitochondrial surface for the fission reaction and induces cell death through mitochondria-dependent pathway in special tissues.^32^ Drp1 activation is regulated by modulating phosphorylation but Ser 637 phosphorylation is proposed to inactivate Drp1.^18, 19^ Our work showed that Rg5 inhibited Drp1 activation and prevented mitochondrial fission by enhancing phosphorylation of Drp1 at Ser 637 in a manner dependent on Akt. Because mitochondrial integrity is essential for HK-II binding to mitochondria, it was convincible that Rg5 protected mitochondrial integrity by inhibition of mitochondrial fission, and thereby provided another way to increase mitochondrial HK-II. Drp1 inhibitor Mdivi-1 and gene knockdown of Drp1 protected mitochondrial HK-II, further supporting the conclusion.

Cytosolic HK-II and Drp1 translocate to mitochondria, demonstrating opposite effect on mitochondrial function. We found that inhibition of Drp1 activation increased mitochondrial HK-II. It was therefore worthwhile knowing if the binding of HK-II to mitochondria prevented the recruitment of Drp1 to mitochondria. 2-DG is a modified glucose molecule that cannot undergo further glycolysis in cell. 2-DG mimics glucose 6-phosphate accumulation in cells and is usually used to induce HK-II detachment from mitochondria, whereas insulin is considered to increase HK-II binding to mitochondria. In resting cells, Drp1 is inactive and mainly located in the cytoplasm. In the present study, when mitochondrial HK-II was reduced by 2-DG treatment, Drp1 expression in mitochondria increased. In contrast, insulin treatment inhibited PA-induced Drp1 recruitment to mitochondria. These results strongly suggested that Drp1 and HK-II controlled each other and competed for the binding to mitochondria. Consistent with the reciprocal control, Rg5 inhibited Drp1 recruitment to mitochondria and increased mitochondrial HK-II via Akt activation, demonstrating the double targets to regulated by Akt in mitochondria.

Mitochondrial HK-II detachment and fission impaired mitochondrial integrity, leading to mitochondrial dysfunction in cardiomyocytes. Mitochondria are the main source of ROS production, though increased ROS amount could impair mitochondrial function through oxidative stress. Intracellular calcium accumulation is a resultant effect of altered ionic homeostasis. To re-establish ionic homeostasis, ATP is shifted away from contractile purpose to remove intracellular calcium, and the futile ATP consumption should be the main reason for the impairment of cardiac energy efficiency. Rg5 inhibited ROS production and reduced intracellular calcium accumulation, contributing to improving ionic homeostasis. Mitochondrial HK-II, together with VDAC and the ANT are essential for the maintenance of the permeability barrier of IMM.^11^ mPTP is activated by Ca^2+^ together with phosphate and ROS, and the prolonged opening of mPTP can cause irreversible damage to the heart owing to inner membrane permeabilization, membrane potential dissipation, impaired PTP synthesis and the release of pro-apoptotic proteins.^11^ Consistently, accumulating evidence demonstrates that mitochondrial HK-II can confer protection against ischemic injury in the heart by limiting outer mitochondrial membrane permeabilization.^33^ PA stimulation increased HK-II dissociation from mitochondria with the opening of mPTP and the collapse of *Δψm*, setting cardiomyocytes at a stage susceptible to I/R damage. Indeed, we observed that PA-treated cardiomyocytes are more sensitive to mitochondrial dysfunction and apoptosis. As a result of the protection of mitochondrial function, Rg5 inhibited cytochrome C release and reduced cell death during I/R insult. The preserved mitochondrial integrity and ATP production during I/R insult should be the important reasons for the resistance to cell death. Moreover, we developed cardiac ischemia in mice by injection of isoproterenol to confirm the cardioprotective effect of Rg5 *in vivo*. Isoproterenol-induced lipolysis increases fatty acid availability in the heart, well mimicking the impact of lipid challenge on cardiac metabolism. In the ischemic heart, Rg5 promoted Akt translocation to mitochondria, increased mitochondrial HK-II and prevented Drp1 recruitment, demonstrating its action in the protection of mitochondrial integrity. Similarly, we further confirmed the protective effects of Rg5 on mitochondrial integrity in PA-treated cardiomyocytes. As expected, we observed reduced cell apoptosis in ischemic heart. These results demonstrate that cardiac mitochondrial morphological and functional integrity is essential for resistance to ischemic injury.

In conclusion, the present study demonstrates that Drp1 and HK-II reciprocally control each other, competing for binding to mitochondria. Rg5 protects mitochondrial morphological and functional integrity by regulating HK-II and Drp1 translocation via Akt activation. Moreover, our work suggests that combating mitochondrial fatty-acid oxidation could reduce the susceptibility of the heart to ischemic injury in metabolic disorders.

## Materials and methods

### Materials

Rg5 (purity=98%) was a product of Jiangsu Yongjian Pharmaceutical Technology Co., Ltd. (Jiangsu, China). Tetramethylrhodamine ethyl ester perchlorate (TMRE), isoproterenol, 2-DG, trimetazidine and Mdivi-1 were from Sigma (St. Louis, MO, USA). Cyclosporin A and triciribine were purchased from Apex Bio (Houston, TX, USA). Mito-TEMPOL was purchased from Abcam (Cambridge, MA, USA). These agents were dissolved in dimethyl sulfoxide (DMSO) to obtain stock solutions and the final working concentration of DMSO was <0.1% (v/v). PA (Sinopharm Chemical Reagent wq Co., Ltd. shanghai, China) was dissolved in ethanol to prepare 200 mM stock solution and then was further diluted with medium containing 10% FFA-free BSA at the ratio of 1:19 to obtain a concentration of 10 mM before use. The following items were purchased from the cited commercial sources: anti-phospho-Drp1 (S637) (ab193216), anti-Drp1 (ab184247), anti-PDH E1*α* (Ser 293) (ab177461), anti-CPT1A antibody (ab176320), Donkey Anti-Goat IgG H&L (Alexa Fluor 647) (ab150131), Donkey Anti-Goat IgG H&L (Alexa Fluor 405) (ab175664), anti-Prohibitin (ab75771), Abcam; anti-PDHA1 polyclonal antibody (BS7208), Goat Anti-Rabbit IgG (H+L) (Alexa Fluor 488) (BS13278), anti-GAPDH (AP0063), anti-*β*-Actin (AP0060), Bioworld Technology (St. Paul, MN, USA); anti-Hk-II (#2867 S), anti-p-Akt (Ser 473) (#4060), anti-Akt (#4691), anti-Phospho-Akt Substrate (RXRXXS*/T*) (23C8D2), Cell Signaling Technology (Beverly, MA, USA).

### Cell culture

NRVMs were isolated from 1–2-day-old Sprague-Dawley rat pups, digested with 0.08% collagenase and purified by differential adhesion method. Myocytes were maintained overnight in DMEM supplemented with 10% (v/v^−1^) FBS at 37 ^°^C in a humidified atmosphere of 5% CO_2_. Cells were then serum starved for 2 h prior to intervention.

For PA treatment, cardiomyocytes were incubated with indicated agents for 0.5 h, followed by PA (100 *μ*M) stimulation for 2 h. To mimic ischemic injury in cell, the PA-treated cells were then washed with PBS and exposed to 1% O_2_ with glucose deprivation for 4 h. After that, the cells were re-incubated in a normal DMEM at 37 °C in a 5% CO_2_ humidified incubator for 1 h.

### Isoproterenol-induced myocardial ischemic injury in mice

Male ICR mice (18–22 g), supplied by the Laboratory Animal Center of Nanjing Qinglongshan, were housed in colony cages 12 h light/dark cycles with free access to food and water. The animal care and experimental procedures were approved by Animal Ethics Committee of School of Chinese Materia Medica, China Pharmaceutical University. Rg5 (50 mg/kg) and TMZ (20 mg/kg) were administered orally 1 h before isoproterenol injection (120 mg/kg, subcutaneous) for 2 consecutive days. Animals in the sham groups received equivalent volume of 0.5% CMC-Na solution and saline in the same way. After 24 h of the last injection, blood sample was collected and heart tissue was obtained. For free fatty-acid assays, serum was separated by centrifuging at 3000 rpm for 15 min and then detected with a commercial kit (Jiancheng Bioengineering Institute, Nanjing, China). For western blot analysis, left ventricle regions were quickly frozen in liquid nitrogen followed by storing at −80 °C. For immunofluorescent assay and apoptosis assay, the whole heart was directly fixed in 4% paraformaldehyde or 15% sucrose.

### Measurement of lactate, intracellular calcium, ATP content and NAD+/NADH ratio

NRVMs were treated with Rg5 or other agents at indicated concentrations, and then stimulated by PA or PA combined with hypoxia/reoxygenation. After treatment, the supernatant was used for lactate assay using a commercial kit (Jiancheng Bioengineering Institute). Treated cells were collected, washed with PBS twice and lysed for measurements. Intracellular calcium concentration (Jiancheng Bioengineering Institute), ATP content (Beyotime Institute of Biotechnology, Shanghai, China) and NAD+/NADH ratio (Sigma) were detected with commercial kits respectively according to the manufacturer's protocol.

### OCRs and ECRs measurements

OCRs were measured using the XFe96 Extracellular Flux Analyzer (Seahorse Bioscience, North Billerica, MA, USA). Cardiomyocytes were seeded (10 000 cells/well) and incubated overnight in DMEM supplemented with 10% (v/v^−1^) FBS. One hour before the measurement, cells were incubated with XF medium at 37 °C in a CO_2_-free incubator. The OCRs were detected under basal conditions and after the application of 1 *μ*M oligomycin, 0.5 *μ*M FCCP and 0.5 *μ*M rotenone+0.5 *μ*M antimycin A (XF Cell Q17 Mito Stress Test Kit; Seahorse Bioscience). Each sample was analyzed four times.

Extracellular acidification rates (ECRs) were detected under basal conditions and after the application of 10 mM glucose, 1 *μ*M oligomycin and 50 mM 2-DG (XF Cell Q17 Glycolysis stress test kit; Seahorse Bioscience). Each sample was analyzed four times.

### Molecular docking study

To evaluate the binding potential of Rg5 to Akt protein, *in silico* protein-ligand docking experiment was carried out by using the Autodock program (version 4.2).^34^ The structure of Akt was downloaded from the protein data bank (PDB ID: 1gzk), the crystal water molecules and other small molecules were removed. The Autodock program was then employed to generate an ensemble of docked conformations for Rg5 to Akt protein. We used the genetic algorithm for conformational search. To explore the conformational space of the Rg5 as completely as possible, we performed 100 individual genetic algorithm runs to generate 100 docked conformations. The size of the docking box was properly set to enclose the possible binding pocket. The protein structure was kept fixed during molecular docking.

### Western blot analysis and immunoprecipitation

Cardiomyocytes were lysed in ice-cold RIPA buffer for 5 min, incubated in an ice bath for 45 min and then cleared by centrifugation at 12 000 × g for 20 min at 4 °C. Equal amounts of protein were electrophoresed on SDS-PAGE, transferred to a PVDF membrane and then blocked at room temperature for 2 h. For immunoblotting, the primary antibodies were applied, respectively, at 4 °C overnight, followed by incubation with the secondary antibody at room temperature for 2 h or at 4 °C overnight. Western blot analyses were performed using anti-phospho-Drp1 (S637), anti-Drp1, anti-PDH E1*α* (Ser 293), anti-CPT1A antibody, anti-Prohibitin; anti-PDHA1 polyclonal antibody, anti-GAPDH, anti-*β*-Actin, anti-Hexokinase-II, anti-p-Akt (Ser 473), anti-Akt, anti-Phospho- Akt Substrate (RXRXXS*/T*) specific antibodies. The values of band intensities were detected by enhanced chemiluminescence and quantized by Image-ProPlus 6.0 software.

For immunoprecipitation, NRVMs were treated with Rg5 (10 *μ*M) plus or minus TMZ (10 *μ*M) for 0.5 h before PA stimulation. After treatment cells were washed once with PBS and lysed for 15 min on ice. Cell lysates were then centrifuged at 12 000 × g for 20 min and the soluble fraction was collected. Next, anti-Drp1 antibody or anti-HK-II antibody was immunoprecipitated overnight at 4 °C and then with protein A+G agarose beads (Beyotime Institute of Biotechnology) for another 2 h. After that, the protein A+G agarose beads were washed four times with the lysis buffer. The beads were then boiled in 1% SDS loading buffer for western blotting with the indicated antibodies.

### Immunofluorescence

For the studies in heart from isoproterenol-induced mice, removed heart were fixed with 4% paraformaldehyde for 24 h, dehydrated, embedded in Tissue-Tek O.C.T. Compound (Sakura Finetek, Torrance, CA, USA) and then cut in 8 *μ*m slices. The sections and NRVMs were washed with cold PBS, fixed 4% paraformaldehyde for 20 min. After that, NRVMs were permeabilized with 0.2% Triton X100 and incubated with 5% BSA to block non-specific staining, and then incubated with specific primary antibodies staining (anti-Drp1, anti-HK2, anti-phospho-Akt (Ser 473)) overnight at 4 °C in a humidified chamber. After several washings, the cells were incubated with Alexa Fluor 488-labeled goat anti-rabbit IgG (H+L) antibody, Donkey Anti-Goat IgG H&L (Alexa Fluor 647), Donkey Anti-Goat IgG H&L (Alexa Fluor 405) or Donkey Anti-Goat IgG H&L (Alexa Fluor 488) for 1 h at 37 °C. They were then washed in PBS twice and incubated in DAPI for 15 min at 37 °C. The cells were mounted on a medium and visualized under a confocal scanning microscope.

### The siRNA-mediated gene silencing

H9c2 cells (American Type Culture Collection, Manassas, VA, USA) were maintained in DMEM supplemented with 10% (v/v^−1^) FBS at 37 °C in a humidified atmosphere of 5% CO_2_. To specifically suppress Drp1 and AKT2 expression, H9c2 cells were grown to 80% confluence and then transfected with siRNA duplexes specific for rat AKT2 (sc-108063, Santa Cruz Biotechnology, Santa Cruz, CA, USA), Drp1 (sc-43732, Santa Cruz Biotechnology) or control siRNA (sc-37007) by siRNA transfection reagent (sc-29528), respectively. After transfection, cells were cultured in medium for 48 h. The cells were treated with indicated agents and PA (100 *μ*M) for 2 h and then collected and processed for immunofluorescence microscopy and western blot.

### Cytosol/mitochondria fractionation

Cytosolic and mitochondrial fractions were prepared from NRVMs using Cell Mitochondria Isolation Kit (Beyotime Institute of Biotechnology) by the manufacturer's protocol. In brief, cells were collected by centrifugation at 650 g for 10 min at 4 °C, then the cells were washed twice with cold PBS and resuspended with Lysis buffer. After vortex and incubation on ice for 15 min, the cells were homogenized for 25 stokes and the homogenates were centrifuged at 650 g for 10 min at 4 °C. The supernatants were centrifuged at 11 000 × g for 15 min at 4 °C. The resulting mitochondrial pellets were resuspended with mitochondrial lysis buffer. The supernatants were the cytosolic protein.

### Mitochondrial fission, Δψm and ROS production assay

For mitochondrial fission assay, NRVMs were treated with Rg5, TMZ or Mdivi-1 at different concentrations, and then stimulated with PA or PA followed by hypoxia/reoxygenation. After stimulation, the cells were washed with PBS and incubated with 200 nM Mito Tracker Red CMXRos (Molecular Probes, Thermo Fisher Scientific, San Jose, CA, USA) for 30 min at 37 ^°^C. The structure of mitochondria was viewed or measured using confocal microscopy (Zeiss LSM 700). For *Δψm* assay, treated NRVMs were loaded with the potentiometric dye TMRE (final concentration of 500 nM) for 30 min at 37 °C. TMRE staining was examined by confocal microscopy (Zeiss LSM 700). For intracellular ROS detection, treated NRVMs were loaded with ROS-specific fluorescent probe dyedihydroethidium (Beyotime Institute of Biotechnology) and incubated for 0.5 h and DAPI (Sigma) for 15 min at 37 °C. After washing, cells were fixed in 4% paraformaldehyde for 5 min at 4 °C and viewed by confocal scanning microscopy (Zeiss LSM 700).

### ELISA assay of cytochrome C

NRVMs were seeded in six-well plates, treated with Rg5 (10 *μ*M) or TMZ (1 *μ*M) and stimulated by PA under hypoxia/reoxygenation. Cell cytosol and mitochondria fractions were collected and the concentration of cytochrome C was assayed with commercial enzyme-linked immunosorbent assay (ELISA) kits (R&D, USA).

### Mitochondrial transition pore assay

NRVMs were treated with Rg5 (10 *μ*), TMZ (1 *μ*M), Mdivi-1 (10 *μ*M), cyclosporine A (10 *μ*M) and stimulated by PA combined with hypoxia/reoxygenation. Inomycin (1* μ*M) was added 30 min before detection. Mitochondrial transition pore opening was assayed with Image-IT LIVE Mitochondrial Transition Pore Assay Kit (Molecular Probes) by the manufacturer's protocol. Images were viewed by confocal scanning microscopy (Zeiss LSM 700).

### Apoptosis analysis

NRVMs were treated with Rg5 (10 *μ*M) and TMZ (1 *μ*M) then stimulated with PA (100 *μ*M) under hypoxia/reoxygenation. After stimulation, cells were collected and detected using the AnnexinV-FITC Apoptosis Detection Kit (KeyGEN Biotech Co., Ltd. Nanjing, China). For apoptosis assay in NRVMs, cellular fluorescence was measured using flow cytometry analysis with a FACSCalibur Flow Cytometer (BD Biosciences, San Jose, CA, USA).

### TUNEL assay

Cell death in the myocardium was assayed in heart paraffin section with an *in situ* Apoptosis Detection Kit based on the terminal deoxynucleotidyl transferase-mediated dUTP nick end-labeling (TUNEL) system (Roche Applied Science, Upper Bavaria, Germany).

### Statistical analysis

The data are expressed as the mean±S.D. The significance of differences was analyzed by one-way ANOVA followed by the Bonferroni test. Differences were considered statistically significant at *P*-values of ⩽0.05.
