# Supplementary material for: Ginsenoside Rg5 increases cardiomyocyte resistance to ischemic injury through regulation of mitochondrial hexokinase-II and dynamin-related protein 1
Source: Cell Death Dis. 2017 Feb 23;8(2):e2625–. doi: 10.1038/cddis.2017.43 (PMC5386487; doi:10.1038/cddis.2017.43)
Supplement: Supplementary Information [file cddis201743x1.docx]

**
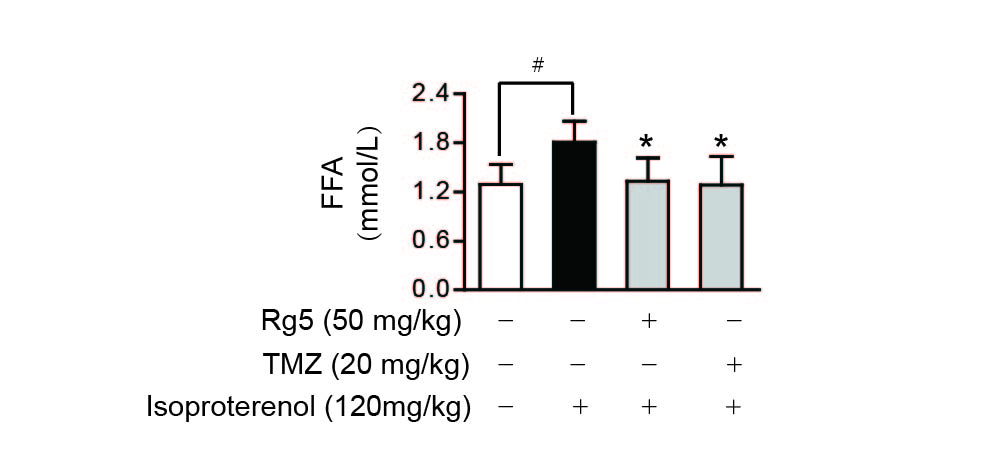
**

**Supplementary Figure** Rg5 reduced blood free fatty acids in isoproterenol-treated mice. Mice were treated with isoproterenol plus or minus oral administration of ginsenoside Rg5 (Rg5) or trimetazidine (TMZ). Content of serum free fatty acids was measured by a commercial kit (n = 8–11). Data are expressed as mean ± S.D.; ^#^*p* < 0.05 *vs.* indicated treatment; **p* < 0.05 *vs.* isoproterenol treatment.
